# Electrochemiluminescence Aptasensor Based on Gd(OH)_3_ Nanocrystalline for Ochratoxin A Detection in Food Samples

**DOI:** 10.3390/bios12121141

**Published:** 2022-12-07

**Authors:** Chunyuan Tian, Minggang Wei, Xiaobin Wang, Qing Hua, Feiyan Tang, Lijun Zhao, Xuming Zhuang, Feng Luan

**Affiliations:** College of Chemistry and Chemical Engineering, Yantai University, Yantai 264005, China

**Keywords:** Gd(OH)_3_ nanocrystals, electrochemiluminescence, aptasensor, ochratoxin A, food detection

## Abstract

In the present study, the electrochemiluminescence (ECL) properties of Gd(OH)_3_ nanocrystals with K_2_S_2_O_8_ as the cathode coreactant were studied for the first time. Based on the prominent ECL behavior of this material and the excellent specificity of the aptamer technique, an ECL aptasensor for the detection of ochratoxin A (OTA) was formulated successfully. Over an OTA concentration range of 0.01 pg mL^−1^ to 10 ng mL^−1^, the change in the ECL signal was highly linear with the OTA concentration, and the limit of detection (LOD) was 0.0027 pg mL^−1^. Finally, the ECL aptasensor was further used to detect OTA in real samples (grapes and corn) and satisfactory results were obtained, which indicated that the built method is expected to be applied in food detection.

## 1. Introduction

Ochratoxin, as a kind of secondary metabolite produced by several species of aspergillus and penicillium, has attracted extensive attention due to its toxicity and serious threat to human health [1,2]. Among these secondary metabolites, ochratoxin A (OTA) is the most common toxic substance that can be found in moldy or fermented agricultural products [3], and it has been proved to be very toxic to the human body, especially to people living in humid and warm environments [4,5]. In addition, OTA can be found throughout the food chain as it contaminates not only food, fruit, and feed, but also coffee, wine grapes, and dried grapes. These OTA-contaminated foods or medicines can cause severe symptoms of either acute or chronic poisoning once someone has consumed them. Therefore, rapid and sensitive detection of OTA in agricultural products and foods is of great significance. Now, the reported detection methods of OTA include high performance liquid chromatography [6], gas chromatography [7], antibody-based enzyme-linked immunosorbent assay [8,9], fluorescence assay [10,11], etc. However, most of these methods may have some disadvantages such as being expensive and time-consuming, requiring bulky equipment, having complicated sample pretreatment steps, as well as some methods only being able to be operated by qualified staff in professional laboratories. Thus, it is urgent to find a simple, rapid, and sensitive detection method.

In order to address these limitations and challenges, various sensors using antibodies or aptamers as capturing probes have focused on the specific recognition and sensitive detection of target small molecules in different substrates such as OTA [12,13,14,15,16]. Among them is the ECL aptasensor, a new technology that combines electrochemistry and chemiluminescence and has received extensive attention. It has the advantages of having a simple operation, strong and stable response, and low background signal [15]. For this, ECL as a high sensitivity detection method has been widely used in many fields [17,18]. It has been reported that aptamers (single-stranded oligonucleotide fragments or short peptides) can be used for the highly selective analysis of OTA [19]. Compared with antibodies, there are some advantages of aptamers, including having a high binding affinity, strong specificity, non-toxicity, etc., that can be applied to cells, proteins, tissues, and small molecules [20,21,22]. By combining the unique advantages of ECL with the specific recognition ability of aptamer, the ECL aptasensor is a general strategy for the sensitive detection of various target molecules.

For the purpose of constructing novel ECL aptasensors, it is very important to select suitable luminescent materials. In recent years, rare earth nanomaterials have attracted extensive attention due to their excellent physical and chemical properties [23,24,25]. For instance, some rare earth compounds have been widely used in high-performance light-emitting devices, catalysts, and other functional materials [26]. It is notable that rare earth hydroxide (Re (OH)_3_) has important application value. Yang and his coworkers [27] tested the biological distribution and toxicity of Gd(OH)_3_ nanorods in vivo and found no toxic effect. They concluded that the nanorods could be used for long-term in-body imaging. In addition, Gd(OH)_3_, as a luminescent material, has been applied in many fields due to its simple synthesis method. However, its application in ECL has not been found yet. Therefore, it is of great significance to use Gd(OH)_3_ as an ECL emitter for OTA detection.

**Scheme 1:**
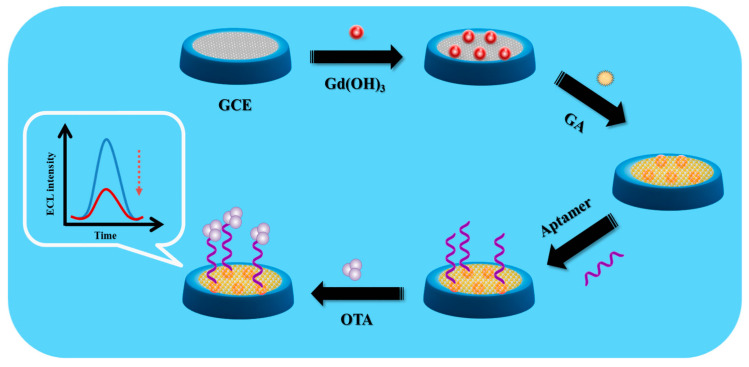
Mechanism of the ECL aptasensor based on Gd(OH)_3_ nanocrystals.

In this work, the Gd(OH)_3_ nanocrystals were synthesized by the glucose-assisted hydrothermal method and its ECL performance was further studied. Based on highly stable and repeatable ECL emission of Gd(OH)_3_ nanocrystals and excellent specificity of the aptamer technique, an ECL aptasensor for OTA detection was constructed. Scheme 1 shows the construction process of an ECL aptasensor. On the one hand, OTA aptamer was coupled with glutaraldehyde (GA) as a crosslinking agent. On the other hand, when the aptamer and OTA were interconnected, ECL intensity would be quenched, which meant high sensitivity and good specificity for OTA detection. The ECL aptasensor was successfully applied to OTA detection of grain and fruit (corn and grapes) with good accuracy. We hope this method can be applied in food detection.

## 2. Materials and Methods

### 2.1. Materials

Gadolinium nitrate hexahydrate (Gd(NO_3_)_3_·6H_2_O) was purchased from Shanghai Aladdin Biochemical Technology Co., Ltd. (Shanghai, China). Glucose, sodium hydroxide (NaOH), and absolute ethanol (C_2_H_5_OH) were purchased from Sinopharm Chemical Reagent Co., Ltd. (Shanghai, China). Glutaraldehyde (GA) was obtained from Xilong Chemical Co., Ltd. (Shantou, China). Ochratoxin A (OTA), chloramphenicol (CHL), penicillin (PE), kanamycin (KAN), neomycin (NEO), and tetracycline (TC) were purchased from Shanghai Sangon Bioengineering Co., Ltd. (Shanghai, China). The base sequence (5′-3′) of the aptamer DNA was 5′-NH_2_-(CH_2_)_6_-GAT CGG GTG TGG GTG GCG TAA AGG GAG CAT CGG ACA-3′.

### 2.2. Apparatus

The size and morphology of the resulting products were captured using an S-4800 scanning electron microscope (HITACHI, Tokyo, Japan). The spectral characteristics of the materials were characterized by Fourier transform infrared (FT-IR; Thermo Electron Corporation, Waltham, MA, USA), ultraviolet–visible absorption spectra (UV–vis; Purkinje General Instrument Co., Ltd., Beijing, China), and fluorescence (F-4700 fluorescence spectrophotometer; HITACHI, Tokyo, Japan), respectively. X-ray diffraction (Rigaku Corporation, Tokyo, Japan) was used to characterize the phase and structure of the prepared nanocomposite. An MPI-E ECL analyzer (Xi’an Remax Analyse Instrument Co., Ltd., Xi’an, China) was used for the ECL measurement.

### 2.3. Synthesis of Gd(OH)_3_

Gd(OH)_3_ nanocrystals were synthesized according to Ref [28]. Briefly, 0.5641 g Gd(NO_3_)_3_·6H_2_O was added into 16 mL ultrapure water to prepare the solution. Next, NH_3_·H_2_O was used to adjust the pH value of the solutions between 3 and 4. A homogeneous solution was prepared by stirring the mixture solution of 14 mL 0.01 M glucose and the above solution for 10 min. The final pH of this homogeneous solution was adjusted to 11 by NaOH. Then, it was transferred to a hydrothermal reaction kettle and reacted at 180 °C for 9 h. The product was cooled down and washed with ethanol and water three times. The final product was dried at 120 °C for 2 h, and the obtained white powder was Gd(OH)_3_ nanocrystals, which was stored in the refrigerator at 4 °C.

### 2.4. Fabrication of ECL Aptasensors

Firstly, the polished glass carbon electrode (GCE) was modified with 5 μL Gd(OH)_3_ solution (1 mg mL^−1^) dissolved by ethanol and dried naturally. Next, 5 μL GA (2%), as a crosslinking agent, was dripped in GCE modified with Gd(OH)_3_ reacting for 2 h. Then, the excess GA was washed by PBS (0.1 M, pH 7.4) solution, the obtained GCE was named Gd(OH)_3_/GCE. Subsequently, the obtained Gd(OH)_3_/GCE incubated in 5 μL adapter solution for 2 h at 37 °C. Due to the presence of GA, the adapter could be fixed to the surface of Gd(OH)_3_/GCE, which was named Apt/Gd(OH)_3_/GCE. Finally, the obtained Apt/Gd(OH)_3_/GCE was used as ECL material and incubated in 100 μL of different concentrations of OTA solutions for 2 h at 37 °C to fabricate a novel ECL aptasensor. It is worth mentioning that after each incubation step, the GCE was washed with PBS (0.1 M, pH 7.4), avoiding non-specific binding, and, finally, dried under nitrogen. Other experimental conditions were as follows: the scanning potential was −2.0~0 V, the scanning speed was 100 mV S^−1^, and the photomultiplier tube was 800 V.

### 2.5. Determination of OTA in Real Samples

In this study, for the purpose of evaluation of the performance of the proposed method, the samples were obtained from local market in Yantai City (corn and grapes). The samples were filtered through a 0.45 μm membrane before further use. Then, they were diluted 10 times with PBS (0.1 M, pH 7.4) and stored at −20 °C for further use.

## 3. Results

### 3.1. Characterization of Gd(OH)_3_

**Figure 1:**
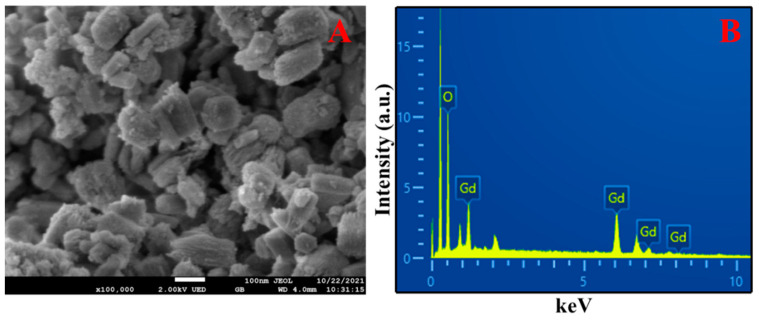
(**A**) SEM image and (**B**) EDS spectrum of Gd(OH)_3_ nanocrystals.

The morphology of the prepared Gd(OH)_3_ nanocrystals was characterized by SEM as shown in Figure 1A. The morphologies of the preparations were columnar (the column length is about 200 nm and the diameter is about 50 nm), evenly dispersed, and have a large, specific surface area. An energy dispersive spectrometer (EDS) was used to verify the element content of Gd(OH)_3_, as shown in Figure 1B. It could be seen that Gd and O elements were present in the nanocrystalline state, which conformed to expectation, providing that the Gd(OH)_3_ nanocrystals were successfully synthesized.

**Figure 2:**
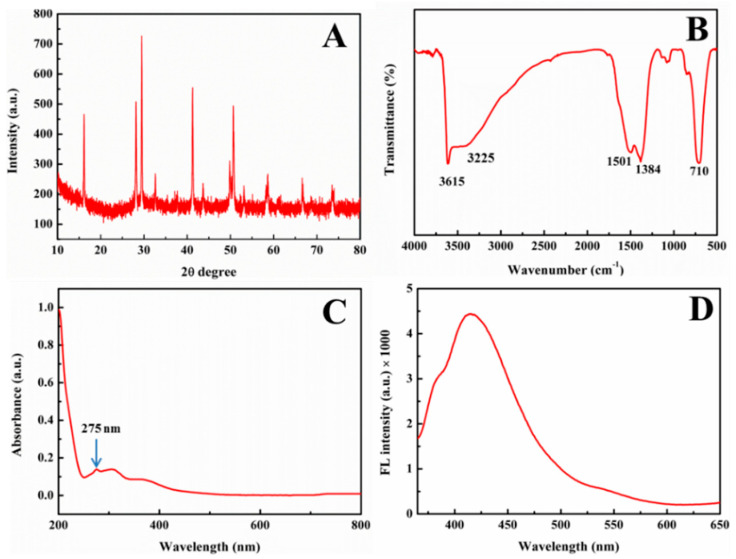
(**A**) XRD spectrum; (**B**) FT−IR spectrum; (**C**) UV−vis absorption spectrum; and (**D**) Fluorescence spectrum of Gd(OH)_3_ nanocrystals.

XRD was then used to further verify the successful synthesis of Gd(OH)_3_ nanocrystals and the results are shown in Figure 2A. As can be seen from the figure, all diffraction peaks were consistent with the Joint Committee on Powder Diffraction Standards card n^0^ 83-2037 [29]. Furthermore, the shape of diffraction peaks of samples was sharp and intense, proving that the prepared Gd(OH)_3_ samples had high crystallinity. Figure 2B shows the image of the FT-IR spectrum. As we know, at 3615 cm^−1^, the O-H stretching vibration peak was very obvious [30], while the peak located at 710 cm^−1^ might be attributed to Gd-O-H bending vibrations [31]. It should be noted that there was a very large peak at 3250 cm^−1^ with a peak of H_2_O, which might be caused by the accuracy of the experimental instrument. In addition, the UV–vis spectra of Gd(OH)_3_ nanocrystals was recorded, which further confirmed the successful preparation of nanocrystals, as shown in Figure 2C. In the figure, there was usually extensive absorption below 400 nm, and this absorption can be attributed to the 4f → 5d transition. In addition, there was a small sharp peak at 275 nm, which was a sign of the transition of Gd (III) from the ground state to the excited state [26]. Figure 2D shows the fluorescence spectra of Gd(OH)_3_ nanocrystals, and it can be seen that the excitation peak was located at 435 nm and the fluorescence color was blue.

### 3.2. Optimizations of Conditions

**Figure 3:**
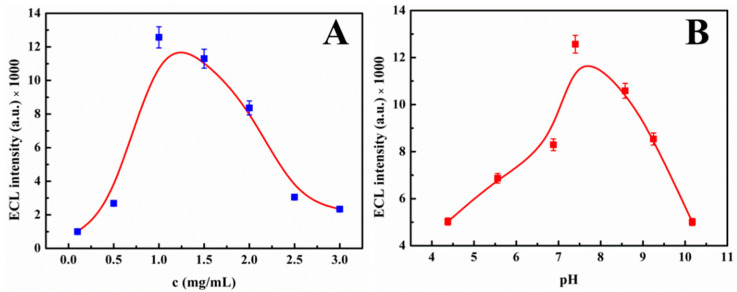
(**A**) Optimization of concentration of Gd(OH)_3_; (**B**) Optimization of pH of K_2_S_2_O_8_ coreaction solution.

In order to obtain the optimal ECL intensity, GCE modified with different concentrations of Gd(OH)_3_ and the pH of the coreaction solution were optimized, as shown in Figure 3. Figure 3A exhibits the concentration optimization of the Gd(OH)_3_ solution (0.1, 0.5, 1.0, 1.5, 2.0, 2.5 and 3.0 mg mL^−1^). With the increase of the concentration of Gd(OH)_3_, ECL intensity increased initially, reaching a peak at 1.0 mg mL^−1^, and then decreased. So, the concentration of Gd(OH)_3_ solution was set as 1.0 mg mL^−1^ in subsequent experiments. Another factor worth considering was the pH of the coreaction solution. K_2_S_2_O_8_ solutions were prepared with different pH (pH = 4.40, 5.60, 6.90, 7.40, 8.60, 9.30, and 10.20). Under the condition of GCE modified with Gd(OH)_3_ (1.0 mg mL^−1^), the ECL intensity was measured (Figure 3B), which suggested that the overloaded Gd(OH)_3_ could enhance the blocking of the electron transfer and inhibition of the diffusion of the coreactant into the electrode, which could hinder ECL. ECL intensity increased initially and then decreased and the pH of the coreaction solution increased gradually. The optimal pH value of the K_2_S_2_O_8_ coreaction solution was selected at pH 7.40. This could be attributed to the pH-dependent surface groups of the Gd(OH)_3_ affecting the ECL reactions. In neutral medium, the Gd(OH)_3_ surface groups have more activity. That is, K_2_S_2_O_8_ with pH 7.40 was selected for subsequent experiments.

### 3.3. Mechanism of ECL Aptasensor

According to the results of the experiments, the possible mechanisms of the ECL sensor based on Gd(OH)_3_ speculated were as follows:

Gd(OH)_3_ + e → [Gd(OH)_3_]^•−^ (1)

S_2_O_8_^2−^ + e → SO_4_^2−^ + SO_4_^•−^ (2)

[Gd(OH)_3_]^•−^ + SO_4_^•−^ → [Gd(OH)_3_]* + SO_4_^2−^ (3)

[Gd(OH)_3_]* → Gd(OH)_3_ + h*v* (4)

On the basis of the aforementioned mechanism, [Gd(OH)_3_]* was ECL emission material, which was formed by the interaction of the K_2_S_2_O_8_ coreaction solution. In the process of ECL measurement, Gd(OH)_3_ obtained electron reduction to [Gd(OH)_3_]^•−^ and S_2_O_8_^2−^ also obtained electron reduction to become a strong oxidant (SO_4_^•−^) on the surface of GCE (Equations (1) and (2)). Meanwhile, [Gd(OH)_3_]^•−^ was oxidized by SO_4_^•−^ into an excited state [Gd(OH)_3_]* (Equation (3)). During the return of the excited state to the ground state, ECL would be generated (Equation (4)).

Under the optimal experimental conditions, OTA was detected using the ECL aptasensor. As shown in Figure S1 (Supplementary Materials), compared with Gd(OH)_3_/GCE, the ECL intensity of Apt/Gd(OH)_3_/GCE (red curve) was decreased slightly, which might be due to the fact that the aptamer hindered the transfer of electrons on the electrode surface to some extent. However, when OTA was incubated, the ECL intensity of OTA/Apt/Gd(OH)_3_/GCE (black curve) was quenched. The main reason for this phenomenon was that the specific binding of OTA and aptamer covered the surface of Gd(OH)_3_/GCE, preventing the reaction between the coreaction solution and the Gd(OH)_3_. The steric hindrance effect produced by OTA might be the main factor that caused the quenching of its ECL intensity.

### 3.4. Performance of ECL Aptasensor for OTA Detection

**Figure 4:**
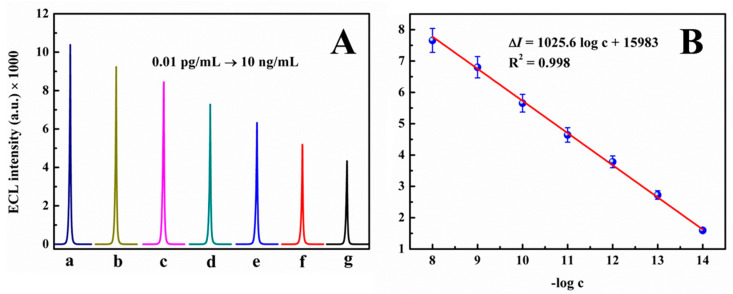
(**A**) ECL response of ECL aptasensor at different OTA concentrations and (**B**) linear relationship of ECL aptasensor to OTA.

Figure 4A shows the ECL intensity recorded after adding OTA solutions with different concentrations (0.01 pg mL^−1^ to 10 ng mL^−1^) to the ECL aptasensor based on Apt/Gd(OH)_3_/GCE. The ECL intensity of Apt/Gd(OH)_3_/GCE decreased gradually with the addition of increasing OTA concentrations. Figure 4B exhibits the standard curves according to the degree of ECL intensity quenching. When the concentration of the OTA solution ranged from 0.01 pg mL^−1^ to 10 ng mL^−1^, the linear equation was ΔI = 1025.6 lgc_(OTA)_ + 15,983 (R^2^ = 0.998) with a low limit of detection (LOD) of 0.0027 pg mL^−1^, indicating the effective detection of OTA at low concentrations (ΔI = I_0_ − I, where I_0_ represents the ECL intensity of Apt/Gd(OH)_3_/GCE and I represents the ECL intensity of OTA/Apt/Gd(OH)_3_/GCE). Moreover, Table S1 summarized some other methods reported to detect OTA previously. Compared with other approaches, the fabricated ECL aptasensor in this study had a lower LOD and a wider detection range, which could meet the growing demand for real sample detection.

### 3.5. Stability, Reproducibility, and Selectivity of ECL Aptasensor

**Figure 5:**
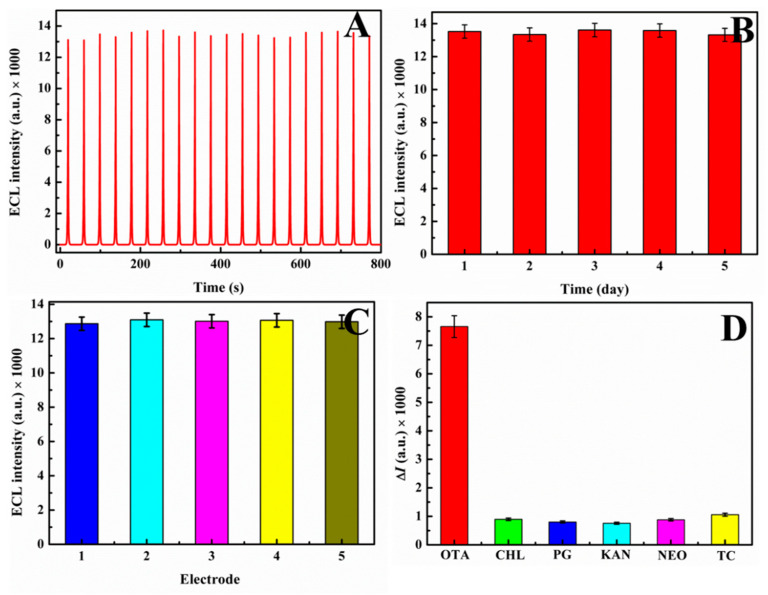
(**A**) The relationship between ECL intensity and time of continuous scanning for 20 cyclic. (**B**) Stability of the ECL aptasensor within 5 consecutive days. (**C**) Reproducibility of the ECL aptasensor with 5 different electrodes. (**D**) Selectivity of the ECL aptasensor for OTA and its analogs at the same concentration.

Stability, reproducibility, and selectivity were important criteria to estimate whether the ECL aptasensor could be applied successfully. Figure 5A shows the ECL behavior of Apt/Gd(OH)_3_/GCE in the presence of S_2_O_8_^2−^ as the coreaction. The ECL intensity of Apt/Gd(OH)_3_/GCE remained high and stable, verifying that Apt/Gd(OH)_3_/GCE was an excellent electrode material. The same electrode was selected for the stability experiment (Figure 5B). After five days of experimenting, the ECL intensity of the electrode has hardly changed, which could prove Apt/Gd(OH)_3_/GCE had favorable stability. Under the same experimental conditions, the reproducibility of the ECL aptasensor was also tested using five electrodes, the results are shown in Figure 5C. It can be seen that the ECL intensity of the five selected electrodes changed insignificantly, implying that the ECL aptasensor had excellent reproducibility.

The good selectivity of this ECL aptasensor was a prerequisite for practical application. Firstly, five kinds of solutions (10 ng mL^−1^ CHL, PG, KAN, NEO, and TC, respectively) were prepared. Next, Apt/Gd(OH)_3_/GCE was incubated in the above solutions and the ECL intensity was recorded. The results are shown in Figure 5D. ΔI of the target OTA was changed obviously compared to other substances, proving that the ECL aptasensor could recognize OTA accurately, while other substances could not interfere with OTA determination.

### 3.6. Detection of OTA in Real Samples

Corn and grapes were selected as the real samples to verify the application of the ECL aptasensor. However, OTA was not found in these samples. Then, the recovery experiment, conducted by adding standard samples, was carried out with the addition of three-level OTA, as shown in Table 1. The recovery rate of OTA was 97.76~102.1%, and the relative standard deviation (RSD) was 1.5~3.6%. The accuracy of the aptasensor established in this work was verified by using high-performance liquid chromatography (HPLC) Ultimate, and OTA was not detected in the actual samples. Then, aliquots of a standard solution of OTA were added into the actual samples and the concentration was determined by the proposed method and the HPLC Method (*n* = 3). The presented statistical studies in Table S2 proved the good precision and accuracy of the proposed method. Thus, the ECL aptasensor was satisfactory in terms of precision and accuracy.

## 4. Conclusions

In this paper, we demonstrated a promising OTA aptasensor based on the co-immobilization of the aptamer of OTA and Gd(OH)_3_ nanocrystalline onto the carbon glass electrode (Apt/Gd(OH)_3_/GCE). The Gd(OH)_3_ nanocrystalline were synthesized by a glucose-assisted hydrothermal method. Based on highly stable and repeatable ECL emission of Gd(OH)_3_ and the excellent specificity of the aptamer technique, the present aptasensor exhibited high sensitivity with a low detection limit, wide linear range, good reproducibility, and high stability. Additionally, this aptasensor has a potential application to assay the OTA in the food samples such as corn and grapes. The aptasensor provided a new method for OTA detection and expanded the application of rare earth oxides in food detection.

## Figures and Tables

**Table 1:** OTA recovery tests in real samples by the ECL aptasensor.

Samples	Added (ng/mL)	Found (ng/mL)	Recovery (%)	RSD (%)
	10	10.2	102.1	1.8
Grape	1.0	1.01	101.4	2.1
	0.1	0.099	99.52	1.7
	10	10.1	100.7	3.6
Corn	1.0	0.98	98.56	2.8
	0.1	0.097	97.76	1.5

## Data Availability

Not applicable.

## Supplementary Materials

The following supporting information can be downloaded at: https://www.mdpi.com/article/10.3390/bios12121141/s1, Figure S1: ECL intensity versus potential for Gd(OH)_3_/GCE, Apt/Gd(OH)_3_/GCE and OTA/Apt/Gd(OH)_3_/GCE; Table S1 [12,32,33,34]: Comparison of different methods for the detection of OTA; Table S2: Determination of OTA in corn by the proposed method and the HPLC Method (*n* = 3).
