# Supplementary material for: Electrochemiluminescence Aptasensor Based on Gd(OH)3 Nanocrystalline for Ochratoxin A Detection in Food Samples
Source: Biosensors (Basel). 2022 Dec 7;12(12):1141. doi: 10.3390/bios12121141 (PMC9775045; doi:10.3390/bios12121141)
Supplement: Supplementary file 1 [file biosensors-12-01141-s001.zip › biosensors-2032091-supplementary.pdf]

Supporting Information

Electrochemiluminescence Aptasensor Based on $Gd(OH)_3$ Nanocrystalline for Ochratoxin A Detection in Food Samples

Chunyuan Tian, Minggang Wei, Xiaobin Wang, Qing Hua, Feiyan Tang, Lijun Zhao, Xuming Zhuang * and Feng Luan *

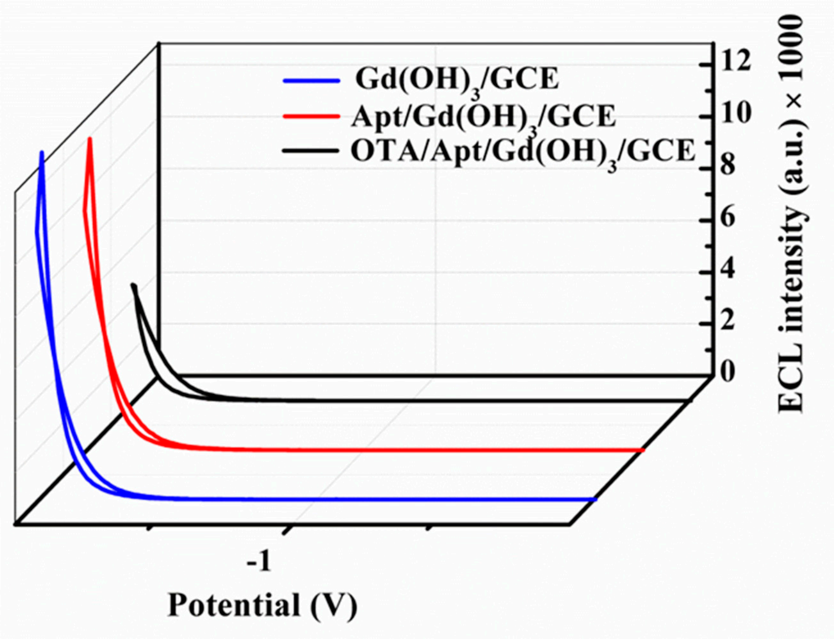

Figure S1. ECL intensity versus potential for $Gd(OH)_3$/GCE, Apt/$Gd(OH)_3$/GCE and OTA/Apt/$Gd(OH)_3$/GCE.

Table S1. Comparison of different methods for the detection of OTA.

Methods	Linear range (ng mL^{-1})	LOD (pg mL^{-1})	Ref.
Fluoresence	0.001~0.05	0.2	[1]
EC[a]	0.01 ~ 10^4	110	[2]
PEC[b]	10^{-4}~200	0.0035	[3]
EC	0.03~10	13.3	[4]
ECL	10^{-5}~10	0.0027	This work

[a]Electrochemistry [b]Photoelectrochemistry.

Table S2. Determination of IM in fish by the proposed method and the HPLC Method (n=3).

Sample	Added (ng/mL)	Amount found by ECL method (ng/mL)	Amount Found by HPLC Method (ng/mL)	Relative error (%)
Corn	1.0	0.98	0.96	2%

References

[1] Zhang: J.; Xia, Y. K.; Chen, M.; Wu, D. Z.; Cai, S. X.; Liu, M. M.; He, W. H.; Chen, J. H. A fluorescent aptasensor based on DNA-scaffolded silver nanoclusters coupling with Zn(II)-ion signal-enhancement for simultaneous detection of OTA and AFB1. *Sensor. Actuat. B-Chem.* **2016**, *235(1)*,79-85.

[2] Barthelmebs, L.; Hayat A.; Limiadi, A. W.; Marty, J. L. Noguer, T. Electrochemical DNA aptamer-based biosensor for OTA detection, using superparamagnetic nanoparticles. *Sensor. Actuat. B-Chem.* **2021**, *156(2)*, 932-937.

[3] Feng, J. H.; Qian, Y. R.; Cheng, Q.; Ma, Y. M.; Wu, D.; Ma, H. M.; Ren, X., Wang, X. Y.; Wei, Q. A signal amplification of p DNA@Ag_2S based photoelectrochemical competitive sensor for the sensitive detection of OTA in microfluidic devices. *Biosens. Bioelectron.* **2020**, *168(15)*, 112503.

[4] Zhu, C. X.; Liu, D.; Li, Y. Y.; Ma, S.; Wang, M., You, T.Y. Hairpin DNA assisted dual-ratiometric electrochemical aptasensor with high reliability and anti-interference ability for simultaneous detection of aflatoxin B1 and ochratoxin A. *Biosens. Bioelectron*. **2021**, *175(15)*, 112654.
